# Ethanolamine plasmalogens derived from scallops stimulate both follicle-stimulating hormone and luteinizing hormone secretion by bovine gonadotrophs

**DOI:** 10.1038/s41598-022-20794-4

**Published:** 2022-10-06

**Authors:** Hiroya Kadokawa, Miyako Kotaniguchi, Shiro Mawatari, Risa Saito, Takehiko Fujino, Shinichi Kitamura

**Affiliations:** 1 Faculty of Veterinary Medicine, Yamaguchi University, Yamaguchi-shi, Yamaguchi-ken, 1677-1 Japan; 2 International Polysaccharide Engineering (IPE) Inc., Laboratory of Advanced Food Process Engineering, Organization for Research Promotion, Osaka Metropolitan University, 1-2, Gakuen-cho, Nakaku, Sakai, Osaka 599-8570 Japan; 3 Institute of Rheological Functions of Food, Hisayama-machi, Fukuoka, 811-2501 Japan; 4 Laboratory of Advanced Food Process Engineering, Organization for Research Promotion, Osaka Metropolitan, University, 1-2 Gakuen-cho, Naka-ku, Sakai, 599-8531 Japan

**Keywords:** Endocrine system and metabolic diseases, Animal physiology

## Abstract

Brain ethanolamine plasmalogens (EPls) are the only known ligands of G-protein-coupled receptor 61, a novel receptor that stimulates follicle-stimulating hormone (FSH), but not luteinizing hormone (LH), secretion by bovine gonadotrophs. We hypothesized that the recently developed neuroprotective EPls extracted from scallop (*Pecten yessoensis*) (scallop EPls) could stimulate FSH secretion by gonadotrophs. To test this hypothesis, bovine gonadotrophs were cultured for 3.5 days and treated with increasing concentrations of scallop EPls. FSH secretion was stimulated by all tested concentrations of scallop EPls (*P* < 0.05). Surprisingly, LH secretion was stimulated by both 0.5 (*P* < 0.05) and 5 (*P* < 0.01) ng/mL of scallop EPls. To clarify the important differences between bovine brain and scallop EPls, we utilized two-dimensional liquid chromatography–mass spectrometry, which revealed 44 peaks, including 10 large peaks. Among them, eight were scallop-specific EPl molecular species, occupying approximately 58% of the total area percentage of scallop EPls. Almost all large peaks contained 4, 5, or 6 unsaturated double bonds in the carbon chain at the sn-2 position of the glycerol backbone. Our results showed that EPls from scallops, lacking pituitary glands, stimulated both FSH and LH secretion by bovine gonadotrophs.

## Introduction

Gonadotrophs in the anterior pituitary (AP) secrete gonadotropins, luteinizing hormone (LH) and follicle-stimulating hormone (FSH), to regulate reproductive functions in mammals^1^. These cells are controlled by gonadotropin-releasing hormone (GnRH) via the GnRH receptor on the gonadotroph surface. G-protein-coupled receptor 61 (GPR61) is a novel receptor that colocalizes with GnRH receptors in lipid rafts on the gonadotroph surface^2^, along with ethanolamine plasmalogens (EPls), a unique alkenyl-acyl-glycerophospholipid class, the only known ligand of GPR61^3^. In the absence of GnRH, EPls extracted from young (approximately 26 months old), healthy bovine brain, but not aged (approximately 90 months old) bovine brain, strongly stimulate gonadotrophs to secrete FSH^4^. The chemical synthesis of EPl is challenging, with only one chemosynthetic EPl being commercially available. Using a cultured AP cell model prepared from post-pubertal (approximately 26 months old) Japanese Black heifers, we have recently reported that GPR61 found in bovine gonadotrophs binds the chemosynthetic EPl, activates the cytoplasmic Smad and ERK pathways, and stimulates FSH and LH secretion^5^. Therefore, EPls may be involved in age-related infertility in gonadotrophs via GPR61. Similar to that of humans, the fertility of cows decreases with age^6^; therefore, further studies on EPls are necessary to address age-related infertility.

EPls contain a fatty alcohol bonded to the glycerol backbone at the sn-1 position with a vinyl-ether bond and a fatty acid bonded to the sn-2 position with an ester bond^7^. Using two-dimensional liquid chromatography-mass spectrometry (2D LC–MS), we have previously reported that bovine brain extract contains at least 20 molecular EPl species based on the various possible combinations of fatty alcohols and acids^4,8^. Among them, upon comparing extracts from old cow brains (approximately 90 months old) with those from young heifers (approximately 26 months old), the levels of three species exhibited significantly lower ratios (*P* < 0.05) in old brains relative to young brains (C16:0-C20:4 [denoting species containing C16:0 as an sn-1 side chain and C20:4 as an sn-2 side chain], C16:0-C22:4, and C18:0-C18:1), whereas three other species (C16:0-C20:1, C18:1-C20:1, and C18:0-C20:1) showed significantly higher ratios (*P* < 0.05) in old brains relative to young brains^4^. Therefore, age-related differences in brain EPl composition, especially the presence of polyunsaturated long-chain fatty acids at the sn-2 position, may contribute to the age-dependent ability of EPls to stimulate gonadotrophs.

Plasmalogens are highly abundant in the neuronal, immune, and cardiovascular cell membranes^9^. Plasmalogens are required for the proper functioning of integral membrane proteins, lipid rafts, and cell signaling; in addition, they play a crucial role as an endogenous antioxidant and immune modulator^9^. Moreover, previous studies have illustrated the important roles of EPl in neuronal protection in human brains. However, the chemical synthesis of specific EPl molecular species is extremely challenging. Previous studies investigated naturally abundant EPls and found bioactive EPls in marine invertebrates^10,11^. In Japan, EPls extracted from scallop (*P. yessoensis*), a marine invertebrate that lacks a brain and pituitary gland, have recently been utilized in neuroprotection. Oral ingestion of scallop EPls significantly improves cognitive function in patients with Alzheimer's disease and those with Parkinson’s disease^12,13^. Acid hydrolysis and one-dimensional LC–MS of scallop EPls suggested a prevalence of molecular species with either polyunsaturated long-chain fatty alcohols at sn-1 or polyunsaturated long-chain fatty acids at sn-2^13^. Recent studies revealed the beneficial effect of scallop EPl in the brains of mice. Scallop EPls enhance endogenous expression of brain-derived neurotrophic factor (Bdnf) in the hippocampus and promote neurogenesis associated with learning and memory in mice^14^. Furthermore, they enhance the recruitment of the CREB transcription factor onto the murine Bdnf promoter region through the upregulation of ERK-Akt signaling pathways in neuronal cells^14^. A recent randomized, double-blind, placebo-controlled trial revealed that orally administered scallop EPls alleviated negative mood states and sleep problems and enhanced mental concentration^15^. Therefore, scallop EPl is the most effective EPl naturally available in Japan. However, no studies have evaluated the effect of the same scallop EPl on FSH and LH secretion from gonadotrophs.

It is difficult to collect human brain samples; the pituitary gland size of laboratory animals is too small for appropriate analysis. In contrast, the size of the whole bovine pituitary gland (at least 4 cm along the rostrocaudal axis, 3 cm along the lateral axis, and 4 cm along the vertical axis) facilitates easy differentiation of the anterior lobe (the hard, light brown or pink portion) from the posterior and intermediate lobes (soft, brown portion) following sagittal dissection along the midline^16^. Individually cultured bovine AP cell models (i.e., non-pooled), have been used in previous studies to evaluate the effect of various hormones, including insulin-like growth factor I, kisspeptin, estrogens, anti-Müllerian hormone, and EPl^4,5,17–21^. In this study, we tested the hypothesis that the same scallop EPls (same origin and methods of purification by same scientists)^12–15^ could stimulate FSH secretion by gonadotrophs. Subsequently, we used 2D LC–MS for obtaining deeper insights into the EPl molecular species present in scallops.

## Results

### EPl stimulates LH as well as FSH secretion by gonadotrophs

Highly purified EPls were prepared from the same species of scallops obtained from the same sea area using a previously reported method^22^. Alkyl-acyl phosphatidylethanolamines were digested by phospholipase A1 and removed during EPl purification. G*Power 3 for windows^23^ was used to estimate the required number of samples with an alpha-error probability of 0.05 and a statistical power of 0.95. We prepared AP cells from healthy post-pubertal heifers (25.7 ± 0.4 months old; n = 6) and cultured them for 3.5 days. We incubated the cells with 0, 0.05, 0.5, 5, or 50 ng/mL (final medium concentration) scallop EPls, and utilized highly purified brain EPls obtained from young Japanese Black heifers (approximately 26 months old; details reported previously^4^) as controls. The medium samples were harvested 2 h after culture for FSH and LH assays.

**Figure 1:**
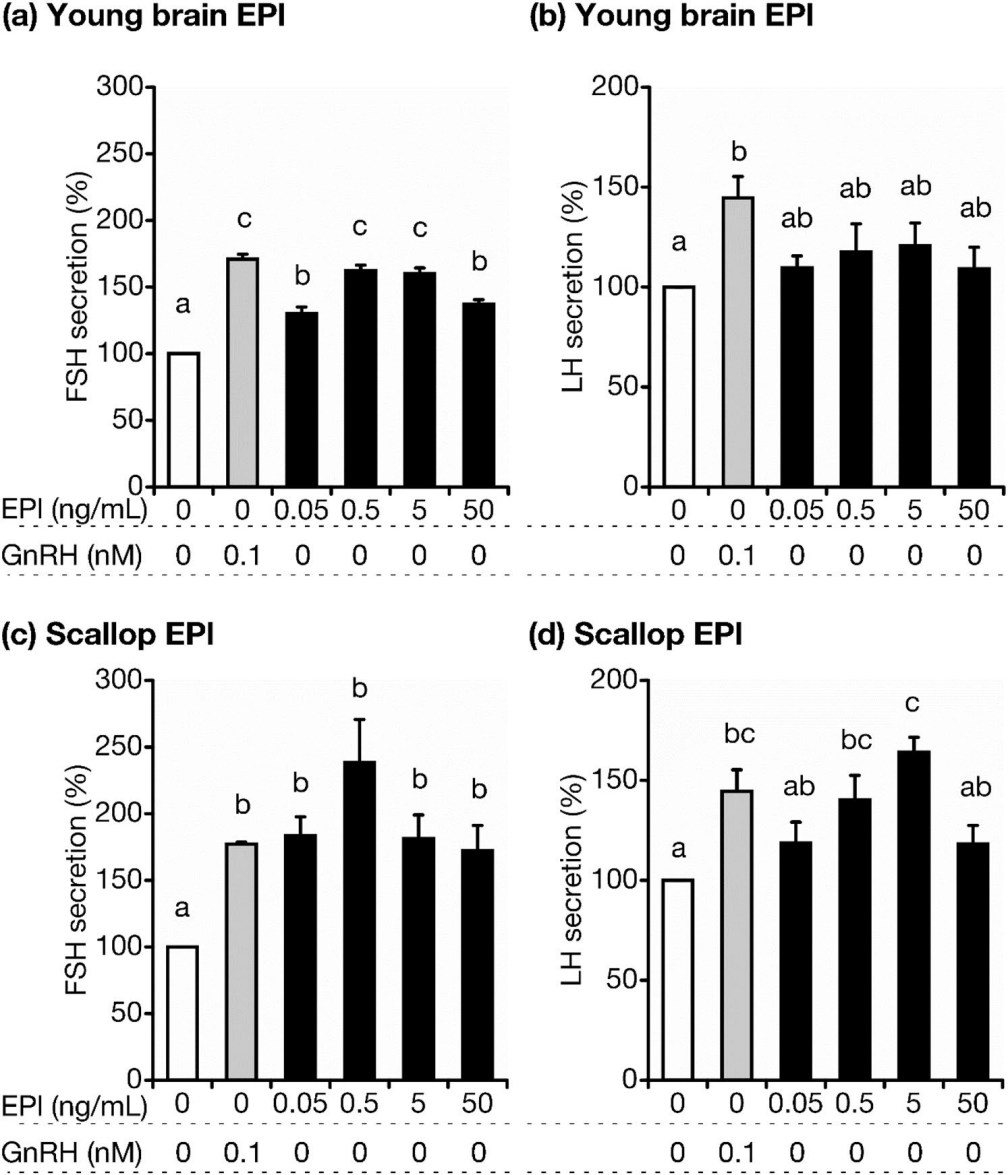
Effects of various concentrations of young heifer brain-derived EPls (**a**, **b**) or scallop-derived EPls (**c**, **d**) in media lacking GnRH on FSH (**a, c**) and LH (**b, d**) secretion by cultured AP cells. FSH and LH concentrations in control cells (cultured in a medium lacking EPls and GnRH) were averaged and set as 100%. The mean LH or FSH concentrations in each treatment group were expressed as percentages of the control value. Bars are labeled with different letters (a, b, and c) to indicate different stimulatory effects (*P* < 0.05; details of *P*-values are presented in Supplementary Tables S1–S4 online). Bars labeled with the same letter indicate a similar stimulatory effect. Statistical analysis was conducted using the Tukey–Kramer test. AP, anterior pituitary; scallop EPl, scallop-derived ethanolamine plasmalogen; GnRH, gonadotropin-releasing hormone; FSH, follicle-stimulating hormone; LH, luteinizing hormone.

All tested concentrations of control EPls stimulated FSH (Fig. 1a) but not LH (Fig. 1b) secretion (statistical values are provided in Supplementary Tables S1 and S2 online). Meanwhile, all tested concentrations of scallop EPls also stimulated FSH secretion (Fig. 1c; statistical values are provided in Supplementary Table S3 online). Surprisingly, 0.5 and 5 ng/mL scallop EPls also stimulated LH secretion (Fig. 1d; statistical values are presented in Supplementary Table S4 online).

### Purity of EPl obtained from scallop and bovine brain

**Figure 2:**
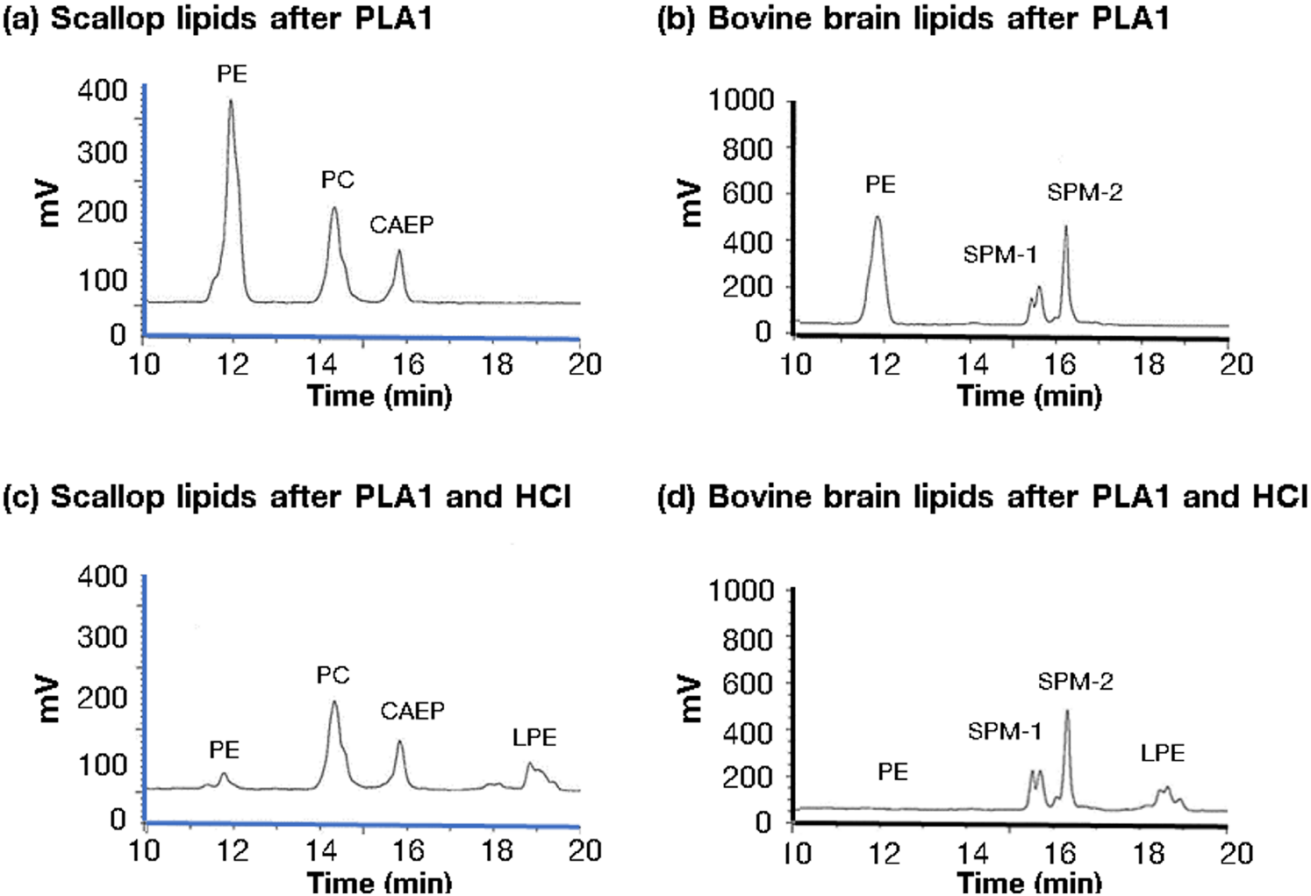
Chromatograms depicting HPLC profiles of scallop- or bovine brain-derived EPl, using the HPLC-ELSD system. Ether phospholipids from scallop (**a**) or bovine brain (**b**) after phospholipase A1 hydrolysis. Ether phospholipids from scallop (**c**) or bovine brain (**d**) after phospholipase A1 hydrolysis and HCl hydrolysis. CAEP, ceramide aminoethylphosphonate; ELSD, evaporative light scattering detector; HCl, hydrogen chloride; HPLC, high-performance liquid chromatography; LPE, 1‑palmitoyl‑2‑hydroxy‑sn‑glycero‑3‑phosphoethanolamine; mV, milli-voltage; PC, 1,2‑dipalmitoyl‑sn‑glycero‑3‑phosphocholine; PE, 1,2‑dipalmitoyl‑sn‑glycero‑3‑phosphoethanolamine; PLA1, phospholipase A1; SPM: sphingomyelin.

We analyzed the scallop and bovine brain EPls after phospholipase A1 hydrolysis and HCl hydrolysis, using a HPLC-Evaporative Light Scattering Detector (ELSD) system. Each sample was analyzed in triplicate. Figure 2 illustrates a representative HPLC profile of scallop and bovine brain EPls. In the scallop and bovine brain samples, 92.6 ± 0.5% and 99.2 ± 0.1% of the total ether glycerophospholipids were alkenyl-acyl-ethanolamineglycerophospholipids (EPl); the remaining 7.4 ± 0.5% and 0.8 ± 0.1% were alkyl-acyl-ethanolamineglycerophospholipids, respectively.

### EPl molecular species in scallop EPl

**Figure 3:**
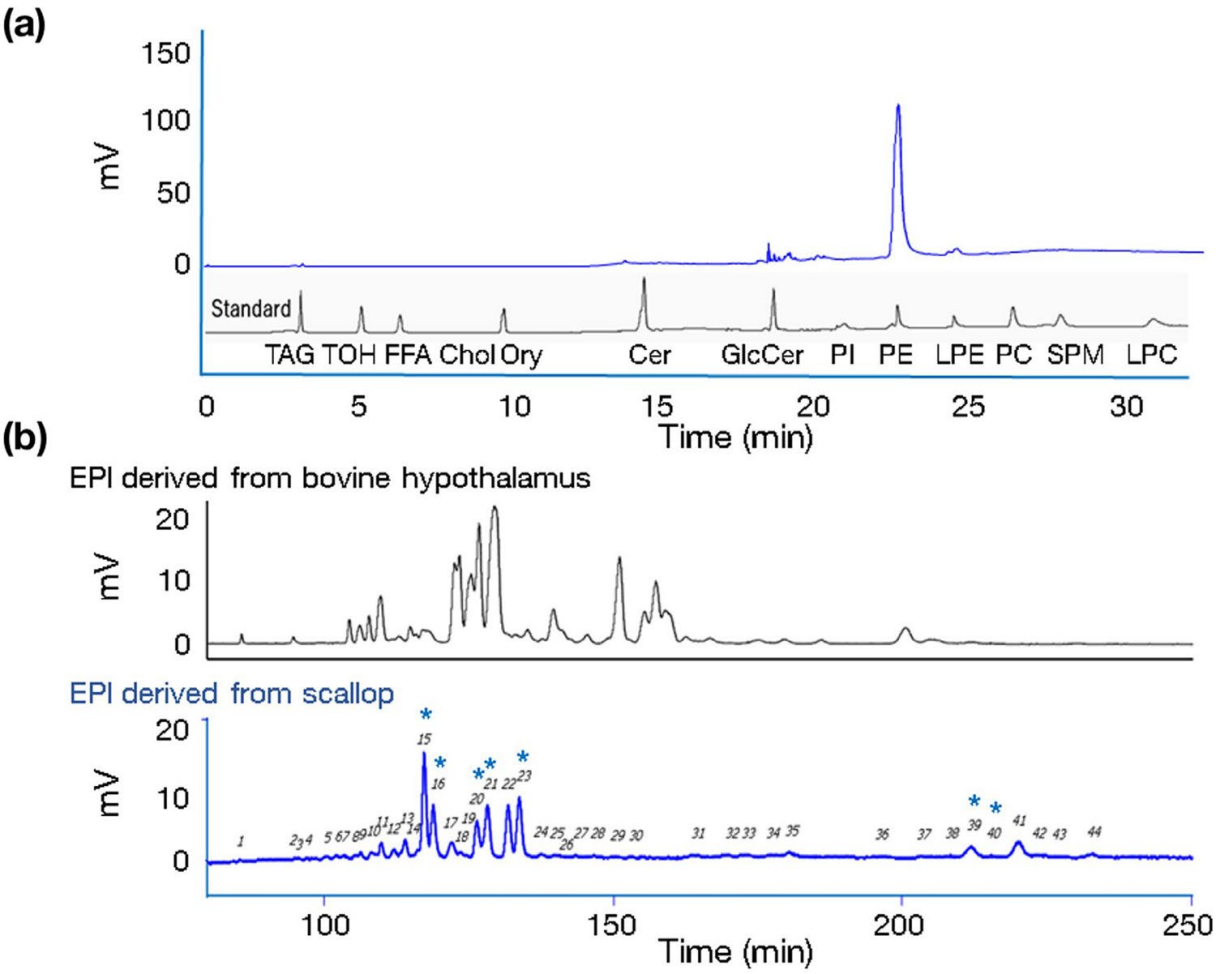
Chromatograms depicting examples of HPLC profiles of scallop-derived EPl. (**a**) Scallop-derived EPls were analyzed using the 2D LC–MS system. The chromatograms depict an example of a first-dimensional HPLC (normal-phase HPLC and a charged aerosol detector) profile of the extracted EPl-rich lipids and 13 lipid standard compounds. (**b**) The lower figure depicts an example profile of second-dimensional HPLC separation (reverse-phase HPLC and a charged aerosol detector) of the EPls molecular species in the phosphatidylethanolamine fraction of scallop EPls, eluted from the first-dimensional HPLC column with 44 peaks. The peaks correspond to the components listed in Table 2. The upper figure depicts second-dimensional HPLC separation of the EPls molecular species in the phosphatidylethanolamine fraction of the heifer brain (details published previously^8^), for comparison. The blue asterisks indicate identifiable EPls molecular species unique to the scallop and absent in the bovine brain (details are presented in Table 2). 2D LC–MS, two-dimensional liquid chromatography-mass spectrometry; HPLC, high-performance liquid chromatography; mV, milli-voltage; scallop EPl, scallop-derived ethanolamine plasmalogen; TAG, tripalmitin; TOH, D-α-tocopherol; FFA, palmitic acid; Chol, cholesterol; Ory, cycloartenyl ferulate; Cer, ceramide; GlcCer, glucosylceramide; PI, phosphatidylinositol; PE, 1,2‑dipalmitoyl‑*sn*‑glycero‑3‑phosphoethanolamine; LPE, 1‑palmitoyl‑2‑hydroxy‑*sn*‑glycero‑3‑phosphoethanolamine; PC, 1,2‑dipalmitoyl‑*sn*‑glycero‑3‑phosphocholine; SPM, sphingomyelin; LPC, 1‑palmitoyl‑2‑hydroxy‑*sn*‑glycero‑3‑phosphocholine.

We analyzed scallop EPls using a 2D LC–MS system. Each peak sample was analyzed in triplicate, and molecular species were identified based on both peak time and *ms*–*ms* profiles. First-dimensional high-performance liquid chromatography (HPLC) comprised normal-phase HPLC and a charged aerosol detector. Figure 3a illustrates an example of a first-dimensional LC profile of scallop EPls and 13 standard lipid compounds. As listed in Table 1, the most prevalent major lipid class was EPls, followed by lysophosphatidylethanolamine.

**Table 1:** Comparison of the peak area ratio of each lipid class to the total peak area of all lipids in scallop-derived EPls.

Lipid class	Area (%)^a^	
	Mean	SEM
EPls	91.91	0.15
Lysophosphatidylethanolamine	2.92	0.05
Free fatty acid	0.28	0.02
Phosphatidylcholine	0.28	0.09
Others	4.61	0.22
Total	100.00	

^a^ Ratio of the peak area of each lipid class to the total peak area.

Triplicate analyses were performed for each lipid.

EPl, ethanolamine plasmalogen; SEM, standard error of the mean.

Subsequently, we analyzed the EPl molecular species using second-dimensional reverse-phase HPLC separation and a charged aerosol detector. Figure 3b presents a sample profile of the EPl molecular species in scallops, exhibiting 44 peaks. For comparison, Fig. 3b also presents a chromatogram of the second-dimensional, reverse-phase HPLC separation of EPls derived from the brain of fertile young heifers (26 months old), as previously described^4^. Table 2 presents the details of the 32 identified EPl molecular species. We compared the molecular species present in scallop EPls to those in bovine brains^4^. This was determined only for molecular species with an area percentage of more than 2.0%. Among the 32 EPl molecular species, 8 peaks (13, 15, 16, 20, 21, 23, 39, and 41) were unique to scallops, while 2 (17 and 22) were present in both scallops and the heifer brain. Scallop-specific EPl molecular species occupied 57.67% of the total area percentage of scallop-derived EPls.

**Table 2:** Composition and comparison of EPl molecular species in scallop-derived EPls.

Peak no	RT (min)	*m/z*^a^	Identified molecular species^b^		Area (%)^d^		Presence in bovine brain^e^
			1st	2nd	Mean	SEM	
1	85.31	810.60	UID ^c^		0.19	0.01	
2	95.03	738.50	UID ^c^		0.16	0.01	
3	95.34	722.70	UID ^c^		0.25	0.01	
4	97.73	748.60	UID ^c^		0.42	0.05	
5	100.38	722.60	16:0–20:5		0.73	0.05	
6	101.90	748.60	18:1–20:5		0.73	0.00	
7	103.42	724.60	UID ^c^		0.73	0.02	
8	105.90	736.63	17:0–20:5		0.68	0.08	
9	106.15	748.60	16:0–22:6		0.96	0.03	
10	107.96	736.60	17:0–20:5		1.42	0.11	
	107.96	774.60	18:1–22:6				
11	109.72	750.60	UID ^c^		2.39	0.02	
12	111.85	776.60	UID ^c^		1.75	0.09	
	111.85	762.60	17:0–22:6				
13	113.91	750.60	18:0–20:5		3.18	0.06	**Absent**
14	116.51	764.64	UID ^c^		1.48	0.17	
15	117.25	750.60	18:0–20:5		12.67	0.15	**Absent**
16	118.80	776.60	20:1–20:5		7.87	0.07	**Absent**
17	121.92	752.60	UID ^c^		3.37	0.08	
	121.92	776.60	18:0–22:6				Present
18	123.43	778.60	UID ^c^		1.07	0.06	
19	124.31	764.60	19:0–20:5		0.62	0.00	
20	126.37	776.60	18:0–22:6		5.64	0.08	**Absent**
21	128.15	802.60	20:1–22:6		9.41	0.10	**Absent**
22	131.65	752.60	18:0–20:4		7.80	0.05	Present
	131.65	778.60	UID ^c^				
23	133.60	804.60	UID ^c^		9.96	0.12	
	133.60	778.60	20:1–20:4				**Absent**
24	137.59	788.70	UID ^c^		1.30	0.08	
	137.59	754.60	UID ^c^		1.30	0.08	
25	140.03	780.70	UID ^c^		0.84	0.03	
26	140.94	766.70	19:0–20:4		0.48	0.06	
	140.94	792.70	UID ^c^				
27	143.31	778.70	18:0–22:5	20:0–20:5	0.72	0.04	
28	145.74	804.70	20:1–22:5		1.10	0.04	
29	150.26	780.70	UID ^c^		0.55	0.07	
30	152.97	806.70	UID ^c^		0.37	0.02	
31	163.88	756.70	18:1–20:1	20:1–18:1	1.14	0.15	
32	169.62	756.70	20:1–18:1	18:0–20:2	0.94	0.08	
	169.62	756.70	18:1–20:1	18:0–20:2			
33	172.33	782.20	20:1–20:2		1.01	0.04	
34	177.12	756.70	18:0–20:2		0.86	0.04	
35	180.09	782.70	20:1–20:2		1.60	0.01	
36	196.38	758.70	18:0–20:1		0.60	0.01	
37	203.35	758.70	18:0–20:1		1.13	0.07	
38	207.55	758.70	18:0–20:1		1.00	0.10	
39	211.77	784.60	20:1–20:1		3.79	0.07	**Absent**
40	215.70	758.70	18:0–20:1		0.73	0.08	
41	220.02	784.60	20:1–20:1		5.15	0.04	**Absent**
42	223.92	786.70	UID ^c^		1.17	0.10	
43	226.34	810.70	20:1–22:2		0.65	0.05	
44	232.69	786.70	UID ^c^		1.42	0.08	
				**Total**	100.00		

^a^ *m/z*: mass to charge ratio, identified as the molecular ion [M + H] + .

^b^ Identified molecular species: denoted as the carbon chains at the sn-1 position and fatty acids at the sn-2 position.

^c^ Molecular species not identified.

^d^ Ratio of the peak area of each EPl molecular species to the total peak area.

^e^ Molecular species present in scallop EPls compared to those in the bovine brains in a previous study. ^8^. This was determined only for molecular species with an area percentage of more than 2.0%.

EPl, ethanolamine plasmalogen; SEM, standard error of the mean; RT, retention time.

The top four most abundant EPl molecular species were 18:0–20:5 (peak 15), 20:1–20:4 (peak 23), 20:1–22:6 (peak 21), and 20:1–20:5 (peak 16), all unique to scallops. All molecular EPl species with an area percentage of more than 2.0%, except for peaks 39 and 41, contained 4, 5, or 6 unsaturated double bonds in the carbon chain at the sn-2 position of the glycerol backbone.

## Discussion

Fertile heifer brain EPls strongly stimulate FSH secretion in bovine gonadotrophs, in the absence of GnRH, via cytoplasmic signaling pathways^4,21^. In this study, scallop EPls stimulated FSH secretion by bovine gonadotrophs. The effect of heifer brain EPls on LH secretion was weak, which is similar to our observations in previous studies^4,21^. Therefore, we did not expect that scallop EPls would stimulate LH secretion in bovine gonadotrophs. However, our results suggest that scallop-specific EPl molecular species may stimulate LH secretion. Lysophosphatidylethanolamine, which lacks an acyl group at the sn-2 position, does not affect FSH and LH secretion^21^. We have previously proposed that the carbon chain at the sn-2 position is crucial for the regulation of gonadotropic secretion^4^. Our current results indicated that 4, 5, or 6 unsaturated double bonds in the carbon chain at the sn-2 position of the glycerol backbone may be essential for stimulating FSH and LH secretion.

The 2D LC–MS system revealed eight peaks of scallop-specific EPl molecular species. A previous study using 1D LC–MS system reported that the predominant fatty acids of EPl species were 20:5 [eicosapentaenoic acid (EPA)] and 22:6 [docosahexaenoic acid (DHA)] at the sn-2 position of the glycerol moiety in marine foodstuffs, whereas major EPl species in land foodstuffs were 20:4.^11^, which is consistent with our results. Although only scallop EPls were used in this study, other marine invertebrates possess EPA or DHA-rich EPls^10,11^. Previous studies have reported the biological effects of EPls derived from mussel (*Mytilus edulis)*, sea cucumber (*Cucumaria frondosa*), and ascidian (*Halocynthia roretzi*) on the brain^24–26^. Therefore, further studies are required to clarify the EPl in other marine invertebrates.

We used the same scallop EPls^12–15^ from the Sea of Okhotsk in northern Japan. We repeated the same experiment to obtain similar results of the stimulation effect using different batches of scallop EPls and different lots of AP cells (data not shown). We used the previously established bovine AP cell model^4,5,17–21^ and observed that the stimulation of FSH and LH secretion from bovine gonadotrophs was indeed attributed to the significant effect of EPls, and not the bias of using bovine gonadotrophs. Scallop farming is most active in the Sea of Okhotsk compared with other areas. The amount of polyunsaturated long-chain fatty acids is higher in scallops in the Sea of Okhotsk than in those in other areas (unpublished data of one of the co-authors of this study, Dr. Fujino, obtained by personal communication). Although we were unable to explain the difference in the amounts of polyunsaturated long-chain fatty acids in scallops between different areas, the first criterion for scallop selection was obtaining them from cold water. Another criterion was selecting scallops that were clean enough to be eaten raw. Thus, the amount of polyunsaturated long-chain fatty acids and degree of hygiene may be important factors for showing the biological effects. However, we could not use other scallop EPls in different areas. Therefore, further studies are warranted to determine if other scallop EPls stimulate FSH and LH secretion. Furthermore, the species used here are taxonomically distant from one another.

The 2D LC–MS system was used in this study for analytical purposes; we could not elute each EPl molecular species separately. Therefore, we did not evaluate the effect of each individual species on FSH and LH secretion. However, scallop-specific EPl molecular species comprised 57.67% of total scallop-derived EPls. Therefore, a 2D LC–MS system, with which each EPl molecular species can be eluted, should be further developed to evaluate the effect of each EPl molecular species on FSH and LH secretion.

Approximately 75% of GPR61-positive cells in the cattle AP gland are gonadotrophs^2^. Although the precise function of GPR61 remains unclear, GPR61-deficient mice reportedly exhibit hyperphagia-associated obesity^24^. GPR61 has also been implicated in type 2 diabetes^25^. Therefore, scallop EPls may affect food intake and body weight via GPR61 in non-gonadotroph AP cells.

In this study, the 50 ng/mL scallop EPls exhibited a weaker stimulatory effect on FSH than the 0.5 ng/mL scallop EPls, as well as a weaker stimulatory effect on LH than 5 ng/mL scallop EPls. Previously, we reported that excess GnRH (> 1 nM) exhibited a weaker stimulatory effect on LH secretion than 0.1 or 1 nM GnRH in the same system of cultured bovine AP cells^26^. The same mechanism may explain why excess scallop EPls exhibited a weaker stimulatory effect.

Old age is associated with decreased fertility in cows^6^. The hypothalamic–pituitary–gonadal axis has a highly conserved anatomy in mammals, owing to its essential function in regulating fundamental aspects of physiological homeostasis^7^. Therefore, the results obtained in this study may be applicable to other mammals.

Interestingly, all EPl molecular species, such as C16:0-C20:4, C16:0-C22:4, and C18:0-C18:1, prevalent in young heifer brains were absent in scallop EPls. Therefore, further studies are warranted to determine the phylogenetic distribution of these EPl molecular species, especially whether they are unique to land mammals. The pituitary gland was only developed during vertebrate evolution and is, therefore, absent in invertebrates^27,28^. The presence of polyunsaturated fatty acids in scallop EPls in this study supports the results previously obtained by acid hydrolysis followed by one-dimensional LC–MS^22^. The scallop EPls in this study were from farmed scallops in the Sea of Okhotsk in northern Japan; thus, the unique EPl molecular species may be biochemical adaptations under severe conditions in cold water^29^. Although scallops have no hypothalamus, various peptides of the GnRH family are secreted from their ganglia for direct control of steroidogenesis and spermatogonial proliferation via receptors on the plasma membrane^30–32^. Further studies are required to determine the role of EPls in reproduction in various species.

In a recent literature review of 23 previous studies^33^, blood FSH concentration in human females approaching menopause decreased in 21 studies, while the remaining reported an increase. However, it is possible that other factors, besides EPl (e.g., estradiol and prolactin), may contribute to controlling blood FSH levels in older human females.

This study focused on the recently discovered GnRH-independent, EPl, and GPR61-dependent FSH and LH secretion. To the best of our knowledge, there have been no reports of another type of plasmalogen, e.g., choline plasmalogen, is mediated by a receptor or pathway to induce a biological mechanism. However, we did not evaluate the effect of choline plasmalogens on FSH and LH; hence, there remains the possibility of choline plasmalogen having some biological effects.

We used the AP cell models derived from sexually matured healthy heifers as non-pooled in this study, consistent with the previous studies, to evaluate the effect of various hormones using a similar number of cattle^4,5,17–21^. We obtained sufficient numbers of gonadotroph cells from a single bovine AP gland. In a previous study using pig AP cells, the authors combined ten pig AP cells to obtain sufficient numbers of gonadotrophs and an adequate LH concentration (reaching the minimum detectable range of the immunoassay)^34^. The usage of pooled AP cells minimizes the effect of individual differences; however, even the smaller ruminant, sheep, can supply a sufficient number of gonadotrophs ^35,36^. Therefore, our data suggest the significant effect of scallop EPl despite individual differences. However, performing in vivo tests on bovines is challenging due to the hydrophobicity of EPls, which must be dissolved in an organic solvent for intravascular administration, to prevent metabolization in tissues. Thus, future studies are required to compensate for the current lack of in vivo data.

Japanese Black female calves reached puberty on average at approximately 12 months of age during the period of linear increase in body weight (approximately 300 kg)^37^. After observing normal estrous cycles, Japanese farmers usually use artificial insemination for postnatal first calving when the body weight is sufficient to avoid dystocia (approximately 500 kg), at 26.5 months of age on average among 2600 Japanese Black heifers^38^. Therefore, we could not obtain heifers at 9 to 15 months of age as the body sizes of the heifers were too small to obtain enough volume of beef from a slaughterhouse; hence, the age of the used heifers matched the definition of sexually mature and young fertile animal with non-biased reproductive status, but without biased changes in the intracellular signaling pathways in gonadotrophs.

Glycerophospholipids are classified into diacyl glycerophospholipids and ether glycerophospholipids. Ether glycerophospholipids are characterized by an alkyl or an alkenyl (a vinyl ether-) linkage at the sn-1 position of the glycerol backbone. Plasmalogens are glycerophospholipids with alkenyl bond^39^. Phospholipase A1 hydrolyses the acyl bond at the sn-1 position of glycerophospholipids; however, it does not act on the alkenyl and alkyl bonds of phospholipids^40^. Therefore, we used phospholipase A1 to remove all the diacyl glycerophospholipids during the preparation of EPls. Unlike alkyl-acyl-phosphatidylethanolamines, EPl can be hydrolyzed by HCl. We observed some residual alkyl-acyl-phosphatidylethanolamines after phospholipase A1 hydrolysis in the scallop and bovine brain EPls. The alkyl-acyl-phosphatidylethanolamines were removed from EPl using second-dimensional reverse-phase HPLC separation^8^. In addition, the electrospray ionization-mass spectrometer differentiates between EPl and alkyl-acyl-phosphatidylethanolamines^8^. Therefore, the data for EPl molecular species are devoid of the noise from alkylacylphosphatidylethanolamines. However, it cannot be denied that alkyl-acyl-phosphatidylethanolamines could influence the stimulation of FSH and LH secretion from gonadotrophs.

In conclusion, our findings supported the hypothesis that scallop EPls stimulate both FSH and LH secretion in bovine gonadotrophs. However, further studies are warranted to extrapolate the suggested beneficial effects with respect to the fertility of land animals.

## Methods

### Ethics statement

All experiments were performed according to the Guiding Principles for the Care and Use of Animals in the Field of Physiological Sciences (Physiological Society of Japan). All experiments involving animals were approved by the Committee of Yamaguchi University (approval number, 301). We complied with the ARRIVE guidelines. All cattle were obtained from contract farmers in western Japan. Following the disaster of bovine spongiform encephalopathy in 2002, all cattle born in Japan are registered at birth in a national database, with an individual identification number. Consumers can obtain information regarding the breed, date of birth, farm of origin, and slaughter by querying the server of the National Livestock Breeding Centre of Japan. We verified the above information in this study. All cattle used in this study were slaughtered to harvest beef according to the regulation of the Ministry of Agriculture, Forestry, and Fisheries of Japan.

For safety, organic solvents were handled inside a fume hood to prevent its inhalation. Insulating gloves were used when handling acids and working with the freezer.

### Preparation of bovine brain EPl

All organic solvents used in HPLC analysis were of HPLC grade and purchased from Wako Pure Chemical Industries, Ltd. (Osaka, Japan). Highly purified brain EPls from fertile young Japanese Black heifers were prepared according to Folch et al.^41^ with minor modifications, and treated with phospholipase A1, as reported previously^4^. Briefly, frozen samples were thawed and homogenized in twice the volume of methanol, and stored overnight at −20 °C. The homogenate was vortexed for re-suspension; 1 mL of the homogenate was transferred to a 10-mL glass centrifuge tube, mixed with 2 mL of methanol and 6 mL of chloroform, and vortexed at room temperature for 10 min. The mixture was centrifuged at 1200 × *g* for 15 min at 25 °C. The upper layer was collected in a glass tube for heat drying (45 ℃) under a gentle stream of N_2_ gas. This extraction was repeated thrice for each sample. After drying, the residue was dissolved in 3 mL of chloroform/methanol (2:1, v/v). This mixture was centrifuged at 1200 × *g* for 15 min at 25 °C; the supernatant was collected into a 10 mL glass centrifuge tube for heat drying (45 ℃) under an N_2_ gas stream. This extraction was repeated two more times for each sample. After drying, 1.5 mL of 0.1 M citric acid buffer (pH 4.5) with or without 100 mg/mL phospholipase A1 (Enzyme commission number 3.1.1.32; 10,000–13,000 units/g; Mitsubishi Kagaku and Foods Co., Tokyo, Japan) was added. The tube was filled with N_2_ gas, capped, and incubated at 45 ℃ for 2 h. When the sample became milky, 10 mL of acetone/hexane (2:1 v/v) was added. The sample was vortexed and centrifuged at 1200 × *g* for 15 min at 25 °C; the upper layer was collected in a glass centrifuge tube for heat drying (45 ℃) under an N_2_ gas stream. This extraction was repeated two more times for each sample. After drying, 10 mL of cold acetone was added, and the sample was vortexed and incubated overnight at -20 °C. The sample was centrifuged at 1200 × *g* for 15 min at 25 °C to remove the supernatant. This washing step was repeated two more times. After the supernatant was removed, the remaining precipitate was dissolved in 10 mL of hexane/acetone (7:3 v/v), and centrifuged at 1200 × *g* for 15 min at 25 °C to collect the supernatant into a glass tube for heat drying (45 ℃) under an N_2_ gas stream. After drying, 6 mL of a hexane/acetone mixture (7:3 v/v) was added, the sample was vortexed, and 0.9 mL of water was added. After shaking, the sample was centrifuged at 1200 × *g* for 15 min at 25 °C; the upper layer was collected into a glass tube of pre-determined weight. This extraction was repeated two more times. After heat drying (45 ℃) under an N_2_ gas stream, the tube was weighed to calculate the weight of the lipids. The tube was filled with N_2_ gas, capped, vacuum-packed, and transported on dry ice to the HPLC-ELSD system and 2D LC–MS system.

### Preparation of scallop EPl

Highly purified EPls from scallops were prepared according to Bligh and Dyer^42^, with minor modifications. Briefly, 1 kg of scallop was homogenized in 3.75 L of methanol/chloroform (2:1, v/v). The samples were incubated for 30 min at room temperature; the sample was mixed with 1 L chloroform followed by the addition of 1 L water. After vigorous mixing, the mixture was centrifuged at 1500 × *g* for 5 min. The chloroform layer (lower layer) was collected into a glass tube, and the remaining upper phase was re-extracted with 1 L chloroform. The combined chloroform layers were dried (45 ℃) under a gentle stream of N_2_ gas.

After drying, 40 mL of 0.1 M citric acid buffer (pH 4.5) with or without 100 mg/mL phospholipase A1 was added to the tube; the cap was filled with N_2_ gas before capping, and the sample was incubated at 45 ℃ for 1 h. The reaction mixture was extracted twice with chloroform and dried. After drying, hexane/2-propanol (3:2, v/v) was used for resuspension. The aliquot tubes were filled with N_2_ gas, capped, vacuum-packed, and transported on dry ice to the HPLC-ELSD system and 2D LC–MS system.

### AP cell collection and culture and analysis of the effects of EPls on FSH and LH secretion

We obtained AP cells from healthy, post-pubertal Japanese Black heifers at the local abattoir, using a previously described method^2,21^. The heifers were in the mid-luteal phase. All cattle were in the luteal phase, as determined by macroscopic examination of the ovaries and uterus^43^; AP cells exhibit the highest LH, FSH, GPR61, and GnRH receptor levels in the luteal phase^2,44^. None of the cattle used in the present study were lactating or pregnant, and none of them had follicular cysts, luteal cysts, or other ovarian disorders^45^.

Enzymatic dispersal of AP cells was performed using a previously described method^26^, and confirmation of cell viability of > 90% was determined via trypan blue exclusion. Dispersed cells were suspended in Dulbecco’s Modified Eagle’s Medium (DMEM), containing nonessential amino acids (Thermo Fisher Scientific, Waltham, MA, USA), 100 U/mL penicillin, 0.05 mg/mL streptomycin, 10% horse serum, and 2.5% fetal bovine serum. Cells (2.5 × 10^5^ cells/mL, total 0.3 mL) were plated in 48-well culture plates and maintained at 37 °C, in a humidified atmosphere of 5% CO_2_, for 3.5 d. Each experiment was performed six times with each of the six different pituitary glands, using four wells per treatment. We supplied recombinant human activin A (final concentration, 10 ng/mL; R&D Systems, Minneapolis, MN, USA) to stimulate FSH synthesis 24 h prior to the tests.

To evaluate the effect of EPls, the initial medium was replaced with 0.25 mL of DMEM containing 0.1% bovine serum albumin and 10 ng/mL activin A and incubated at 37 °C for 2 h. Treatment was performed by adding 0.5 mL of DMEM alone, or 0.5 mL of DMEM containing various concentrations (final concentrations of 0, 0.05, 0.5, 5, or 50 ng/mL) of scallop or heifer brain EPls. After incubation at 37 °C for an additional 2 h, the medium from each well was collected for radioimmunoassay of FSH and LH concentrations, using a previously reported method^2^. These concentrations were selected based on our previous study^4,21^.

### HPLC-ELSD method

The previously reported HPLC-ELSD system^46^ was used for further purification of EPl and for evaluating EPl purity.

The lipid samples were reconstituted with hexane/isopropanol (3:2, v/v) before injection into the HPLC-ELSD system. We used Agilent 1260 system (Agilent Technologies, Tokyo, Japan) equipped with a binary pump, ELSD (Infinity 1290, Agilent Technologies), and column (LiChrospher 100 DIOL, 250 × 3 mm, Merck, Germany). For obtaining EPl, a fraction collector (1260, FCAS, Agilent Technologies) was used for collecting ethanolamine ether lipid peaks from the samples treated with phospholipase A1. Mobile phase A was hexane/2-propanol/acetic acid (82:17:1) with 0.08% triethylamine (TEA), and mobile phase B was 2-propanol/water/acetic acid (85:14:1) with 0.08% TEA. The solvent gradient program was as follows: 0–21 min, B was increased from 4 to 37 (v/v%); 21–25 min, B was increased from 37 to 85%; 25–26 min, A/B (v/v%) 15/85; 29–34 min A/B (v/v%) 96/4. Flow rate was 0.8 mL/min, and column temperature was 50 °C. Chromatographic peaks were detected with ELSD, which was set at gain 6, 50 °C at 3.0 bar for N_2_ gas. The HPLC method can differentiate ether-phosphatidylethanolamines from diacyl-phosphatidylethanolamines and ether-phosphatidylcholine from diacyl-phosphatidylcholine in a single chromatography run. However, the HPLC method cannot distinguish alkenyl-acyl-phosphatidylethanolamines (EPl) from alkyl-acyl-phosphatidylethanolamines.

For distinguishing the alkenyl-acyl-phospholipids in ether phospholipids, the lipid samples treated with phospholipase A1 were further hydrolyzed using 0.1 mL of 36% HCl mixed with 4 mL chloroform/methanol (2:1, v/v) for 30 min at room temperature. The chloroform layer was collected for drying under N_2_ gas. The lipid samples were reconstituted with hexane/isopropanol (3:2, v/v) before injection into the HPLC-ELSD system. The peak remaining after the HCl hydrolysis was considered to indicate alkenyl-acyl-phospholipids. Sphingolipids are not hydrolyzed by either phospholipase A1 or HCl ^40^. Therefore, we calculated the ratios as follows:

EP to S ratio after phospholipase A1 hydrolysis: ratio of peak area of total ether phospholipids after phospholipase A1 hydrolysis to peak area of sphingolipids after phospholipase A1 hydrolysis.

EP to S ratio after phospholipase A1 and HCl hydrolysis: ratio of peak area of total ether phospholipids after phospholipase A1 and HCl hydrolysis to peak area of sphingolipids after phospholipase A1 and HCl hydrolysis.

For the sphingolipid peak, we used ceramide aminoethylphosphonate for scallop and sphingomyelin for bovine brain. The remaining peaks after HCl hydrolysis were considered as alkenyl-acyl-phospholipids. Therefore, EPL purity is calculated as follows:

$$\begin{aligned} EPl \, purity \, & = \, 100 - \left( {100 \, \times \, \hbox{``}EP \, to \, S \, ratio \, after \, phospholipase \, A1 \, and \, HCl \, hydrolysis{^{\prime\prime}}/} \right. \\&\quad \left. {\hbox{``}EP \, to \, S \, ratio \, after \, phospholipase \, A1 \, hydrolysis} \right)^{\prime\prime} \\ \end{aligned}$$

Each sample was analyzed in triplicate; each relative standard deviation of the retention time and peak area was < 0.05 and 0.05%, respectively.

### 2D LC–MS analysis

Both scallop EPls and heifer brain EPls were analyzed using a novel 2D LC–MS system, as previously described^4,8^. This system consists of, (1) normal-phase HPLC, to separate targeted phospholipids (phosphatidylethanolamines, in this study) from other lipid classes, in the first column (first-dimensional HPLC separation); (2) a switching valve, trapping column, and make-up pump, to trap the target lipid class; (3) reverse-phase HPLC to separate the target lipid classes, in the second column (second-dimensional HPLC separation); (4) a charged aerosol detector and electrospray ionization-mass spectrometer to identify and quantify EPl molecular species. The components of 2D LC–MS system, such as the columns, pumps, autosampler, detectors, electrospray ionization-mass spectrometer, and the software, were the same as those previously reported^4,8^. First-dimensional separation was performed using a YMC-Pack PVA-Sil [250 mm length (L) × 4.6 mm internal diameter (I.D.), 5 μm column; YMC Co. Ltd., Kyoto, Japan]. HPLC separation temperature and flow rate were set to 30 °C and 1.0 mL/min, respectively. The lipid sample was prepared at a concentration of 5 mg/mL in chloroform/methanol (2:1 v/v), and a 0.02 mL aliquot was injected into the 2D-HPLC system. The mobile phases, A, B, and C, were hexane, 2-methoxy-2-methylpropane, and methanol, respectively. The solvent gradient program was as follows: 0–7 min A/B/C (v/v/v%) 88/10/2; 7–12 min A/B/C (v/v/v%) 2/88/10; 12–22 min A/B/C (v/v/v%) 2/28/70; 22–32 min A/B/C (v/v/v%) 2/28/70; 32–35 min A/B/C (v/v/v%) 88/10/2. The separation profile was monitored at 210 nm using a variable-wavelength detector. The trapping column was conditioned with a make-up solvent (water/acetonitrile, 40/60, v/v%) before measurement, and the flow rate of the make-up pump was set to 5.0 mL/min. The temperature of the trapping column was set to 27 °C. When the targeted phospholipid fraction was eluted from the first column, the switching valve was changed to mix the targeted phospholipid fraction with the make-up solvent and trap it in a high-carbon-content octadecyl-silica column (YMC-Pack Pro C18 RS; carbon content: 22%; 30 mm L × 4.6 mm I.D., 5 μm column; YMC Co. Ltd.). Thereafter, the flow channel was switched to the second-dimensional HPLC component. EPls molecular species were separated using a hybrid silica-based column (YMC Triart-C18; 250 mm L × 4.6 mm I.D., 3 μm column; YMC Co. Ltd.) at 40 °C. The mobile phase was acetonitrile/methanol/20 mM ammonium acetate (25/68.5/6.5, v/v/v%) at a flow rate of 1 mL/min. The eluent from the second column was split into a charged aerosol detector and electrospray ionization-mass spectrometer. The acquisition range and N_2_ gas pressure of the charged aerosol detector were 500 pA and 241.3 kPa, respectively. An electrospray ionization-mass spectrometer was used to identify the species (positive ion mode; N_2_ sheath gas flow rate: 5 units; capillary temperature: 250 °C; source voltage: 5 kV; capillary voltage: 30 V; tube lens voltage: 80 V). The data-dependent mode was set up with two scan events: one to collect the full mass spectrum of all ions in the sample (MS range *m/z*: 300–2,000), while the other to collect the tandem MS (MS^2^) spectra of the most intense ions at each time point from the MS spectrum in the scan event. The dynamic exclusion setting was as follows. The repeat count for each ion was set to 3, with a report duration of 10 s, an exclusion list size of 30, and an exclusion duration of 30 s. Collision-induced dissociation was conducted with an isolation width of 4 Da and a normalized collision energy of 35. Each sample was analyzed in triplicate; each relative standard deviation of the retention time and peak area was < 0.05 and 0.93%, respectively.

### Statistical analysis

Data were analyzed using StatView version 5.0 for Windows (SAS Institute, Inc., Cary, NC, USA). The Shapiro–Wilk test or the Lilliefors test was used to evaluate the normality or log-normality of each variable, respectively—all variables were normally distributed. Via Grubb’s test, we verified that there were no outliers for any of the variables. Differences in LH or FSH concentrations were analyzed using the Tukey–Kramer test. The level of significance was set at *P* < 0.05. Data are expressed as means ± standard errors of the mean.

## Supplementary Information

Supplementary Information.

## Data Availability

The datasets of the present study are available from the corresponding author upon reasonable request.
