# Supplementary material for: Ethanolamine plasmalogens derived from scallops stimulate both follicle-stimulating hormone and luteinizing hormone secretion by bovine gonadotrophs
Source: Sci Rep. 2022 Oct 6;12:16789. doi: 10.1038/s41598-022-20794-4 (PMC9537335; doi:10.1038/s41598-022-20794-4)
Supplement: Supplementary file 1 — Supplementary Information. [file 41598_2022_20794_MOESM1_ESM.pdf]

Supplementary Material

Ethanolamine plasmalogens derived from scallops stimulate both follicle-stimulating hormone and luteinizing hormone secretion by bovine gonadotrophs

Hiroya Kadokawa, Miyako Kotaniguchi, Shiro Mawatari, Risa Saito, Takehiko Fujino, Shinichi Kitamura

Supplementary Table S1. Details of P-values presented in Figure 1a

	Control	GnRH	0.05	0.5	5	50
Control	-	0.001	0.001	0.001	0.001	0.001
GnRH	0.001	-	0.001	0.596	0.362	0.001
0.05	0.001	0.001	-	0.001	0.001	0.758
0.5	0.001	0.596	0.001	-	0.900	0.001
5	0.001	0.362	0.001	0.900	-	0.002
50	0.001	0.001	0.758	0.001	0.002	-

The P-values were calculated using the Tukey-Kramer test to compare the effects of various concentrations (ng/mL) of heifer brain-derived EPls on FSH secretion from cultured anterior pituitary cells.

EPl, ethanolamine plasmalogen; FSH, follicle-stimulating hormone; GnRH, gonadotropin-releasing hormone.

Supplementary Table S2. Details of P-values presented in Figure 1b

	Control	GnRH	0.05	0.5	5	50
Control	-	0.037	0.900	0.785	0.665	0.900
GnRH	0.037	-	0.159	0.413	0.537	0.150
0.05	0.900	0.159	-	0.900	0.900	0.900
0.5	0.785	0.413	0.900	-	0.900	0.900
5	0.665	0.537	0.900	0.900	-	0.900
50	0.900	0.150	0.900	0.900	0.900	-

The P-values were calculated using the Tukey-Kramer test to compare the effects of various concentrations (ng/mL) of heifer brain-derived EPls on LH secretion from cultured anterior pituitary cells.

EPl, ethanolamine plasmalogen; LH, luteinizing hormone; GnRH, gonadotropin-releasing hormone.

Supplementary Table S3. Details of P-values presented in Figure 1c

	Control	GnRH	0.05	0.5	5	50
Control	-	0.049	0.027	0.001	0.033	0.077
GnRH	0.049	-	0.900	0.180	0.900	0.900
0.05	0.027	0.900	-	0.280	0.900	0.900
0.5	0.001	0.180	0.280	-	0.242	0.122
5	0.033	0.900	0.900	0.242	-	0.900
50	0.077	0.900	0.900	0.122	0.900	-

The P-values were calculated using the Tukey-Kramer test to compare the effects of various concentrations (ng/mL) of scallop-derived EPls on FSH secretion from cultured anterior pituitary cells.

EPl, ethanolamine plasmalogen; FSH, follicle-stimulating hormone; GnRH, gonadotropin-releasing hormone.

Supplementary Table S4. Details of P-values presented in Figure 1d

	Control	GnRH	0.05	0.5	5	50
Control	-	0.022	0.695	0.048	0.001	0.713
GnRH	0.022	-	0.382	0.900	0.655	0.364
0.05	0.695	0.382	-	0.569	0.0186245	0.900
0.5	0.048	0.900	0.569	-	0.473	0.550
5	0.001	0.655	0.019	0.473	-	0.017
50	0.713	0.364	0.900	0.550	0.017	-

The P-values were calculated using the Tukey-Kramer test to compare the effects of various concentrations (ng/mL) of scallop-derived EPls on LH secretion from cultured anterior pituitary cells.

EPl, ethanolamine plasmalogen; LH, luteinizing hormone; GnRH, gonadotropin-releasing hormone.
